# Diagnosis of GATA2 haplo-insufficiency in a young woman prompted by pancytopenia with deficiencies of B-cell and dendritic cell development

**DOI:** 10.1186/s40364-018-0127-x

**Published:** 2018-03-21

**Authors:** Allen Sanyi, David L. Jaye, Cecilia B. Rosand, Amanda Box, Chandrakasan Shanmuganathan, Edmund K. Waller

**Affiliations:** 1 Debusk College of Osteopathic Medicine, Lincoln Memorial University, Harrogate, TN 37752 USA; 2 Department of Pathology, Emory University School of Medicine, Atlanta, GA 30322 USA; 3 Winship Cancer Institute, Emory University, Atlanta, GA 30322 USA; 4 Division of Bone Marrow Transplant, Aflac Cancer and Blood Disorders Center, Children’s Healthcare of Atlanta, Emory University School of Medicine, Atlanta, GA 30322 USA; 5 Department of Hematology/Oncology, and Pathology, Bone Marrow and Stem Cell transplantation, Emory University School of Medicine, 1365B Clifton Road, Suite B5119, Atlanta, GA 30322 USA

**Keywords:** GATA2 deficiency, Bone marrow failure, B-cell deficiency, Allogeneic transplant

## Abstract

**Background:**

GATA2 deficiency presents with a spectrum of phenotypes including increased susceptibility to viral and bacterial infections, multi-lineage cytopenias, aplastic anemia, leukemic transformation and lymphedema. Allogeneic transplantation is only curative therapy for GATA2 deficiency, but is associated with significant treatment related morbidity and mortality. Given the spectrum of clinical presentation, accurate diagnosis of GATA2 deficiency is necessary to identify patients early in their disease course when allogeneic bone marrow transplantation may be of clinical benefit.

**Case presentation:**

In this report, we present a GATA2 mutation diagnosed in 23-year-old woman presenting with pancytopenia, recurring oral blisters, fatigue and chronic pain. We describe markedly low levels of mature B-cells in the blood and bone marrow and the absence of detectable blood dendritic cells with normal serum immunoglobulin levels and normal numbers of marrow plasma cells. She was ultimately diagnosed with GATA2 haplo-insufficiency due to a GATA2 germ-line mutation and underwent a successful allogeneic bone marrow transplant from a 10/10 HLA matched unrelated donor.

**Conclusions:**

The case illustrates the diagnostic difficulties in identifying GATA2 deficiencies and the importance of family history and genetic testing. GATA2 plays an important role in B-cell and dendritic cell development, and decreased numbers of those cells is a characteristic feature that should prompt consideration of GATA2 deficiency in a patient with pancytopenia. Maturation of B-cells to long-lived plasma cells is relatively unaffected in GATA2 deficiency. Allogeneic stem cell transplantation can correct immune-deficiencies and prevent leukemic transformation in patients with GATA2 deficiency.

## Background

GATA2 is a zinc finger transcription factor that is a critical regulator of gene expression in hematopoietic cells [1]. GATA 2 haplo-insufficiency is a rare germline condition that results from heterozygous mutations in the promoter region or coding sequence of the GATA2 gene which can either lead to protein dysfunction or uniallelic reduced GATA2 mRNA transcription. The consequence of reduced levels of functional GATA2 protein include a spectrum of hematological, infectious, pulmonary and dermatologic conditions that typically present in children or young adults with clinical manifestations of multi-lineage cytopenia, immunodeficiency with increased susceptibility to viral and non-tuberculous mycobacterial infections, pulmonary alveolar proteinosis and peripheral lymphedema, with decreased B-cells, monocytes, dendritic cells and NK cells in the blood and marrow [2, 3]. The relative deficit of GATA2 during hematopoiesis contributes to the development of aplastic anemia, myelodysplasia, and acute leukemia [2, 3]*.* The molecular basis for GATA2 deficiency includes a variety of germ-line mutations involving the GATA2 locus on chromosome 3 leading to loss of one of the functional GATA2 genes or reductions in GATA2 mRNA transcription. Homozygous deficiency of GATA2 is embryonically lethal early in gestation in mice [4] while conditional knock-out of GATA2 in adult mice leads to pancytopenia with loss of hematopoietic progenitors and dendritic cells [5]. The nature of germline mutations follows an autosomal dominant pattern with diagnostic confirmation by genetic testing. The protean manifestations in phenotype encompass immune deficiencies and cytopenias, and a broad range of age at presentation (13-74 years). Such manifestations coupled with the rareness of the mutation make GATA2 deficiency difficult to diagnose and illustrate the necessity of a high clinical suspicion [2]. To date, allogeneic bone marrow transplantation (BMT) is the only potentially curative treatment for GATA2 deficiency. Patients with GATA2 deficiency should be offered allogeneic bone marrow transplantation prior to the development of fatal cytopenias, chronic infections due to immune-deficiency, or leukemic transformation. While allogeneic BMT may still be curative in GATA2-deficient patients who develop MDS and AML, the efficacy and safety of transplant is higher if it is performed earlier in the natural history of this complex disorder, prompting consideration of GATA2 deficiency in the differential diagnosis of a patient who presents with cytopenia and immunodeficiency.

In this report, we describe clinical manifestations and treatment of a woman with GATA2 deficiency who presented with a history of pancytopenia, recurrent oral ulcers, fatigue and chronic pain.

## Case presentation

**Fig. 1:**
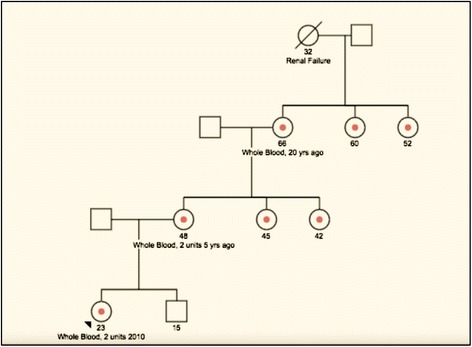
Pedigree of the affected family. Arrow indicates the patient of discussion

A 19-year-old white female presented to a hematology clinic for further evaluation and management of aplastic anemia. At age 9, she began menstruation with heavy bleeding. At age 13, she was found to have low platelet counts around 20 × 10^3^/μL. At age 14, she was diagnosed with mild aplastic anemia based upon a bone marrow biopsy showing a hypocellular marrow without morphological or immunophenotypic evidence for leukemia or myelodysplasia. Her only transfusion history was 2 units of packed red blood cells for worsened anemia related to menorrhagia at age 16 (Hgb of 9.9 g/dL), and she received no specific therapy for aplastic anemia for the next 3 years. In addition to anemia, she complained of frequent sinus and urinary tract infections. She had no history of tobacco use, alcohol consumption, or illicit substance use. A family history revealed a maternal grandmother with a diagnosis of systemic lupus erythematosus, and that both sisters of her maternal-grandmother had anemia with chronically low leukocyte and platelet counts, but no documented immune disorders. A maternal great-grandmother had Charcot-Marie-Tooth syndrome and died at age 32 of renal failure. The patient’s mother, age 48, received 2 units’ whole blood 5 years earlier for treatment of anemia. Additionally, her mother had 2 sisters ages 42 and 45, both of whom had anemia, chronic low blood counts, and autoimmune disease (Fig. 1). There was no history of leukemia in the patient’s family history. There was no clear diagnosis to explain her anemia, thrombocytopenia and leukopenia, and marrow studies that failed to document AML or MDS. She was given empiric supplemental oral iron 325 mg three times a day, vitamin B12 1000 mcg daily, and folate 1 mg daily without any significant change in her symptoms or blood counts. She was then self-referred to our center for further evaluation and diagnosis of her hematological abnormalities.

Upon presentation, physical examination showed a normal appearing Caucasian young woman, with mild bruising to her upper extremities, a normal pulmonary and cardiac exam, normal abdominal exam, with no palpable lymphadenopathy or splenomegaly. Positive clinical findings included diffuse superficial apthous-like ulcers in her mouth, vagina, and nasopharynx. A complete blood count showed a low WBC (2.2 × 103/uL) with absolute neutropenia (absolute neutrophil count of 140 cells/uL), an absolute monocyte of 0.14 × 103/uL, a hemoglobin of 11 g%, RBC count of 3.1 x 10E6/μl with a MCV of 100 fL. She was thrombocytopenic, but transfusion independent, with a platelet count of 29 x 10E3/μl (Table 1). The peripheral blood smear showed pseudo-Pelger Huet forms and rare hypogranular neutrophils while red cell morphology showed anisopoikilocytosis, elliptocytes and dacryocytes. Blood levels of vitamin B12 (295 pg/mL), folate (> 20.0 ng/mL), and copper (162 mcg/dL) were in the normal range, with mild iron deficiency (serum iron 28 mcg/dL, normal range for women 55–160 μg/dL). HLA typing was ordered for possible allogeneic transplant should her hematological parameters worsen and she became transfusion dependent. Her oral ulcers were treated with acyclovir for presumed HSV infection, however, a throat culture for HSV was negative. She was followed clinically for a year with no substantial change in her blood counts but with worsening constitutional symptoms (Table 2).

**Table 1:** Complete blood count and differential table at age 22

Complete Blood Counts and Differential	Values	Reference Range
White Blood Cell Count	2.2 × 10^3^/uL	4 - 10 × 10^3^/uL
Red Blood Cell Count	3.22 × 10^6^/uL	3.93 – 5.22 × 10^6^/uL
Hemoglobin	11 g/dL	11.4 - 14.4 g/dL
MCV	100 fL	79.4 - 94.8 fL
MCH	34.4 pg	25.6 - 32.2 pg
MCHC	34.3 g/dL	30.0 - 36.0 g/dL
Hematocrit	31. 8%	33.3 - 41.4%
Platelet Count	29 × 10^3^/uL	150 – 400 × 10^3^/uL
Neutrophil (Differential)	25%	36 - 75%
Lymphocyte (Differential)	68%	27 - 47%
Absolute Neutrophil	0.14 × 10^3^/uL	0.91 - 5.5 × 10^3^/uL
Absolute Lymphocyte	1.48 × 10^3^/uL	0.65 - 3.05 × 10^3^/uL
Absolute Monocyte	0.14 × 10^3^/uL	0.16 - 0.72 × 10^3^/uL

**Table 2:** Serum immunoglobulin levels

Dates	IgG (620 – 1400)	IgA (80 – 350)	IgM (45 – 250)
6/12/14	1040 mg/dL	77 mg/dL	98 mg/dL
6/5/17	1120 mg/dL	65 mg/dL	70 mg/dL

**Fig. 2:**
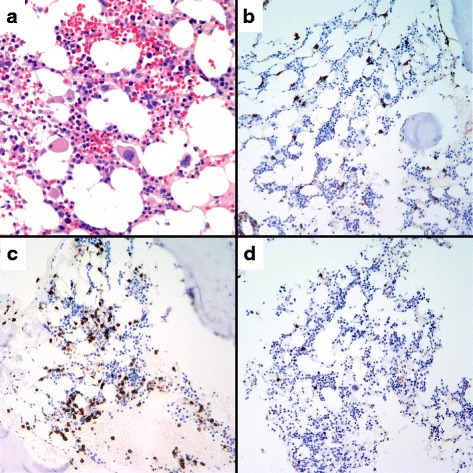
Bone marrow biopsy showing increased fat and decreased cellularity with relative paucity of normal B-cells. **a** H&E (100×). **b** IHC CD138 (200×), occasional plasmacytoid reactive cells. **c** IHC CD3 (200×), numerous small immunoreactive cells. **d** IHC CD20 (200×), scattered small immunoreactive cells

A second bone marrow biopsy performed at age 20 (Fig. 2) showed a hypocellular marrow with mild erythroid and myeloid atypia and atypical megakaryocytes without increased myeloblasts or abnormal cytogenetics. Flow cytometry of the marrow aspirate failed to identify any abnormal cell populations. Immunohistochemistry of the marrow biopsy showed abundant CD3 positive T cells, while CD138 positive plasma cells and CD20 positive B cells each comprised about 1% of nucleated cells She had findings consistent with chronic inflammation/ immune activation as evidenced by a mildly elevated CRP of 10.3 mg/L (normal range 0.3-8 mg/L) and elevated IL-6124 pg/ml (normal value < 5 pg/ml). No obvious infectious etiology was identified. Repeat laboratory testing showed evidence showed decreased CD107a expression on NK cells indicating abnormal degranulation, suggestive of hemophagocytic lymphohistiocytosis (HLH) [6]. Based on persistent cytopenia, and evidence of chronic inflammation and immune activation, low grade MDS, aplastic anemia, immunodeficiency and HLH were the leading differential diagnosis considered. However, serum ferritin was 12 μg/L, not markedly elevated as would be expected for HLH making this diagnosis an unlikely cause of her chronic inflammation or pancytopenia. She was started on a trial of Cyclosporine 200 mg BID (steroids not given) as immunosuppression for treatment of possible aplastic anemia, but this was discontinued after a month due to intolerance and absence of hematological improvement. Additionally, she did not have stigmata of congenital bone marrow failure syndromes such as short stature, skeletal abnormality, or skin hyperpigmentation.

**Fig. 3:**
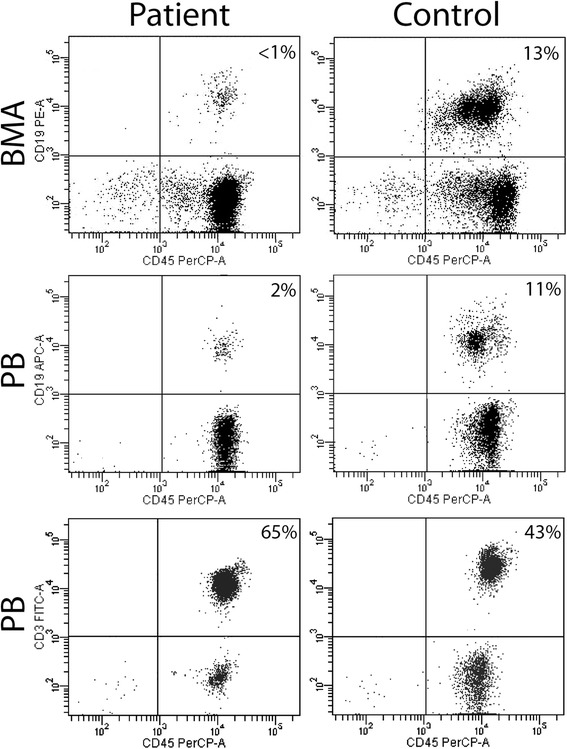
Flow cytometry of bone marrow and blood showed reduced percentages of B-cells with normal frequencies of T-cells. Top row of panels: bone marrow; middle and bottom row of panels: blood. Left panels: blood and bone marrow samples from patient. Right panels: blood and bone marrow samples from a control patient, 5 years status post an allogeneic bone marrow transplant with normal levels of blood lymphocytes and no graft-versus-host disease. Flow plots show antibodies used for staining leukocytes on the x and y axes; percentages listed in the right upper quadrant of each plot show percentage of leukocytes that were CD19+ B-cells (top two rows) or CD3+ T-cells (bottom row)

Evaluation for potential immune defect showed a normal levels of serum immunoglobulins. Immunophenotyping of blood and bone marrow by flow cytometry showed decreased B-cells (2.5% CD19-positive cells), non-detectable plasmacytoid and classical dendritic cells (DC), but normal levels of CD4+ and CD8+ T-cells (Table 3 and Fig. 3). The presence of normal immunoglobulin levels and normal T cell numbers with absence of recurrent or opportunistic infectious ruled out diagnosis of late onset combined T and B cell immune defects and common variable immune deficiency [7]. The presence of peripheral blood monocytopenia with marked B cell lymphocytopenia and absence of DC in a setting of hypocellular marrow, a diagnosis of GATA2 was considered. Polymerase chain reaction based-sequence analysis of *GATA2* revealed a heterozygous missense mutation in Allele 1: c.1061C > T (p.t354 M) that has been previously described for this condition [3]. Although the patient’s parents were not tested, her family history suggested an autosomal dominant pattern of inheritance affecting her mother and mother’s relatives, who appeared to have a mild presentation of the same disorder that affected the patient. With no related HLA-matched sibling or haploidentical donor available, a suitable 10/10 HLA-matched unrelated donor was identified in the NMDP database. After a careful discussion of the natural history of GATA2 deficiency and the risks and benefits of allogeneic transplant, the patient underwent allogeneic BMT from a 10/10 HLA matched unrelated donor following a myeloablative conditioning regimen of busulfan, fludarabine and low dose rabbit ATG [8]. She received post-transplant immunoprophylaxis with methotrexate and tacrolimus and engrafted with neutrophils on day + 18 and platelets on day + 37, achieving 100% donor myeloid and 99% donor T cell chimerism upon variable nucleotide terminal repeat analysis of blood leukocytes on day + 52 post-transplant. Her post-transplant course was uneventful, other than pneumonitis of unknown etiology, cytomegalovirus reactivation with a gastric and duodenal biopsy showing CMV pathogenic effect which has responded to treatment dose valganciclovir and four infusions of IVIG, and low-level EBV viremia which regressed without any specific intervention, all occurring prior to day 100 post-transplant. She did not develop graft versus host disease and achieved transfusion-independence with normal blood counts.

**Table 3:** Blood lymphocyte and dendritic cell subsets over time. pDC = plasmacytoid dendritic cell; mDC = myeloid dendritic cell

Dates	CD3+ T-cells/uL (678-2504)	CD4+ T-cells/uL (500-1500)	CD8+ T-cells/uL (162-1038)	γδ TCR+ T-cells/uL	CD56+ NK cells/uL (45-523)	CD19+ B-cells/uL (96-515)	mDC/uL	pDC/uL
11/2015	1364	751	559	39	103	37	0	0
12/2014	1436	858	520	41	108	47	0	0

## Discussion and conclusions

This case illustrates the need for a heightened clinical suspicion of GATA2 haplo-insufficiency in patients who present with cytopenias and clinical evidence for immunodeficiency. This patient’s initial work-up did not establish a clear diagnosis. Vitamin and mineral deficiencies that may cause pancytopenia were ruled out, and she was treated briefly with immunosuppression for a diagnosis of mild aplastic anemia without clinical improvement. The diagnosis of GATA2 can be particularly challenging due to an inconsistent relationship between genotype and phenotype and unpredictable natural history and age at the time of clinical presentation. The missense mutation detected in this patient is one among many other missense, frameshift, and nonsense mutations that result in GATA2 deficiency [3]. Of note, family members that share the same GATA2 genetic lesion may have different clinical presentations, as was presumably the case with this patient, who had more severe pancytopenia and constitutional symptoms than the other family members with medical histories of mild leukopenia, but were not genotyped. Both inherited and sporadic mutations of the GATA2 gene result in impaired self-renewal of hematopoietic progenitors, which over time progresses to cytopenias that often do not present until adulthood [9]. The risk of infectious complications in GATA2 deficiency is also unpredictable, but frequently increase in the second and third decades [9]. Allogeneic bone marrow transplantation is the only curative therapy for GATA2 deficiency, but is associated with significant treatment related morbidity and mortality. The decision to offer allogeneic HSCT to patients with GATA2 deficiency should be guided by the clinical presentation of the patient, donor availability, and the risk that delaying allo-HSCT until severe clinical manifestations of GATA2 deficiency develop will result in increased mortality.

What are the clinical clues that might have led to an earlier diagnosis of GATA2 deficiency in this or similar patients? In retrospect, the patient had presented with common features of GATA2 deficiency, including pancytopenia with a hypocellular bone marrow, monocytopenia, and a relative deficiency of B cells and DC in her blood and marrow [9–12]. Marrow and blood studies were consistent with a low-grade MDS with mild multi-lineage atypia in the marrow biopsy without increased blasts or cytogenetic abnormalities, and without a need for chronic transfusions. Her biggest complaint was her recurrent apthous ulcers with constitutional symptoms of fever and fatigue that limited her ability to work or attend school. The immunopathological findings in the bone marrow of patients with GATA2 often overlap with those of patients with myelodysplastic syndrome (MDS) since GATA2 deficiency can evolve into MDS and/or AML [3]. Bone marrow biopsies from patients with non-GATA2-related hypo-cellular MDS and GATA2 deficiency may both show presence of focal fibrosis, myeloid dysplasia, and variable numbers of CD34+ blasts, but complex cytogenetic abnormalities are more typical in hypo-cellular MDS than GATA2 deficiency [10, 13]. Both GATA2 deficient and MDS patients may have decreased numbers of CD34 + CD10 + CD20+ hematagones and mature B-cells in the marrow [14], but patients with GATA2 deficiency usually have additional deficiencies of NK cells and monocytes in their marrow, and their plasma cells may express an abnormal CD19+ CD56+ phenotype [10]. While megakaryocytes with dysplastic features are increased in de novo MDS and GATA2 deficiency, the presence of both large and small megakaryocytes with separated and peripheralized nuclear lobes may provide a morphologic clue to an underlying GATA2 deficiency [10]. In this case, bone marrow findings were consistent with low-grade MDS, and the diagnosis of GATA2 deficiency was established after hematological and immunology tests showed deficiencies in numbers of blood monocytes, B-cells and dendritic cells, and GATA2 genotyping established a missense mutation. Thus, a diagnosis of GATA2 deficiency should be considered and appropriate genetic testing undertaken the setting of a patient with a new diagnosis of aplastic anemia, MDS, or immunodeficiency who presents with monocytopenia noted on the peripheral blood smear in, or B lymphopenia and decreased dendritic cells are noted upon immunophenotyping of blood leukocytes, as seen in Fig. 3.

The reduced content of B-cells in the blood and bone marrow in the face of normal serum immunoglobulin levels is notable in this case. GATA2 plays an important role in B-cell and dendritic cell development, and depletion of those cells is a characteristic feature of patients with GATA2 mutation [5, 11, 12]. Novakova et al. noted decreased numbers of immature CD10+ and naïve B cells in pediatric patients with GATA2 mutation, which was reflected in very low level of recombination excision circle (KREC) in blood and bone marrow [12]. Despite significantly reduced numbers of B-cells in blood and marrow, IgG levels were normal in a majority of patients, with increased numbers of plasma cells in their marrow [12]. This patient had mature plasma cells in the bone marrow and normal levels of immunoglobulins despite low numbers of B-cells in the blood and bone marrow (Fig. 3), consistent with published cohort studies [10, 12].

Finally, when considering allo-transplantation, the possibility of GATA2 deficiency in relatives must be considered. As noted in this case, affected family members can have a variable clinical presentation. If a HLA matched or haplo-identical related donor HSCT is being considered, immunological and genetic studies should be performed so as to exclude related with asymptomatic GATA2 genetic abnormalities.

## Abbreviations

AML: Acute myeloid leukemia
BMT: Bone marrow transplant
CBC: Complete blood count
CMV: Cytomegalovirus
DC: Dendritic cell
EBV: Epstein Barr virus
HCT: Hematopoietic Cell Transplantation
HLH: Hemophagocytic lymphohistiocytosis
HSV: Herpes Simplex Virus
IHC: Immunohistochemistry
KREC: Recombination excision circle
MCV: Mean Corpuscular Volume
MDS: Myelodysplastic syndrome
PB: Peripheral blood
